# Anti-IL-20 antibody improved motor function and reduced glial scar formation after traumatic spinal cord injury in rats

**DOI:** 10.1186/s12974-020-01814-4

**Published:** 2020-05-14

**Authors:** Jung-Shun Lee, Yu-Hsiang Hsu, Yi-Shu Chiu, I-Ming Jou, Ming-Shi Chang

**Affiliations:** 1 Division of Neurosurgery, Department of Surgery, National Cheng Kung University Hospital, College of Medicine, National Cheng Kung University, Tainan, Taiwan; 2 Department of Cell Biology and Anatomy, College of Medicine, National Cheng Kung University, Tainan, Taiwan; 3 Institute of Basic Medical Sciences, College of Medicine, National Cheng Kung University, Tainan, Taiwan; 4 Research Center of Clinical Medicine, National Cheng Kung University Hospital, College of Medicine, National Cheng Kung University, Tainan, Taiwan; 5 Institute of Clinical Medicine, College of Medicine, National Cheng Kung University, Tainan, Taiwan; 6 Department of Biochemistry and Molecular Biology, College of Medicine, National Cheng Kung University, Tainan, 704 Taiwan; 7 Department of Orthopedics, E-Da Hospital, I-Shou University, Kaohsiung, Taiwan

**Keywords:** IL-20, SCI, Neuroinflammation

## Abstract

**Background:**

Spinal cord injury (SCI) causes devastating neurological consequences, which can result in partial or total paralysis. Irreversible neurological deficits and glial scar formation are characteristic of SCI. Inflammatory responses are a major component of secondary injury and play a central role in regulating the pathogenesis of SCI. IL-20 is a proinflammatory cytokine involved in renal fibrosis and liver cirrhosis through its role in upregulating TGF-β1 production. However, the role of IL-20 in SCI remains unclear. We hypothesize that IL-20 is upregulated after SCI and is involved in regulating the neuroinflammatory response.

**Methods:**

The expression of IL-20 and its receptors was examined in SCI rats. The regulatory roles of IL-20 in astrocytes and neuron cells were examined. The therapeutic effects of anti-IL-20 monoclonal antibody (mAb) 7E in SCI rats were evaluated.

**Results:**

Immunofluorescence staining showed that IL-20 and its receptors were expressed in astrocytes, oligodendrocytes, and microglia in the spinal cord after SCI in rats. In vitro, IL-20 enhanced astrocyte reactivation and cell migration in human astrocyte (HA) cells by upregulating glial fibrillary acidic protein (GFAP), TGF-β1, TNF-α, MCP-1, and IL-6 expression. IL-20 inhibited cell proliferation and nerve growth factor (NGF)-derived neurite outgrowth in PC-12 cells through Sema3A/NRP-1 upregulation. In vivo, treating SCI rats with anti-IL-20 mAb 7E remarkably inhibited the inflammatory responses. 7E treatment not only improved motor and sensory functions but also improved spinal cord tissue preservation and reduced glial scar formation in SCI rats.

**Conclusions:**

IL-20 might regulate astrocyte reactivation and axonal regeneration and result in the secondary injury in SCI. These findings demonstrated that IL-20 may be a promising target for SCI treatment.

## Background

Spinal cord injury (SCI) causes temporary or permanent spinal cord damage, which results in partial or total paralysis [1]. The irreversible neurological deficits can be devastating to patients and caregivers. The pathophysiology of SCI has been explored in recent decades, revealing a series of complex neuroinflammatory responses (i.e., secondary injury) following the primary mechanical insult and the subsequent formation of glial scar [2–4].

The primary damage is local, segmentally-limited damage to the spinal cord that results in rupture or contusion of axons and development of hemorrhage, ischemia, and edema. The secondary damage considerably expands with further neuronal destruction and reactivation of glial cells [5]. Scar formation by reactive astrocytes in the injured spinal cord subsequently limits secondary damage by providing mechanical stability and restricting infiltration of inflammatory cells. However, the glial scar also impedes the ability of severed axons to regrow and results in persistent neurological dysfunction [6].

Reactive astrocytes expressing glial fibrillary acidic protein (GFAP) demarcate the injury site from healthy tissue by forming a glial scar [7]. The glial scar at the lesion site is considered a major factor that inhibits axon regeneration [8]. In addition to physical inhibition of axonal growth by the glial scar, several other factors inhibit axonal regeneration after SCI. Myelin-associated proteins, such as Nogo-A, myelin-associated glycoprotein (MAG), oligodendrocyte-myelin glycoprotein (OMgp), and Nogo receptor (NgR) signaling, have a central role in the inhibition of axonal regeneration [9, 10]. The extracellular matrix molecule semaphorin 3A (Sema3A) may also inhibit axonal regeneration [11].

Inflammatory responses are a major component of secondary injury and play a central role in the pathogenesis of SCI, which may result in apoptosis of neurons and oligodendrocytes, scar formation, and finally in the reduction of neuronal function. Therefore, it is believed that reducing inflammation could decrease secondary degeneration after SCI. Inflammatory responses that occur after SCI are initiated by resident microglia, followed by peripherally derived immune cells and activated glial cells that proliferate or migrate into the lesion site. Macrophages and microglia contribute to the secondary pathological and inflammatory response, in part through the release of cytokines, tumor necrosis factor (TNF), interleukin (IL)-1, IL-6, and IL-10 [12]. Transforming growth factor-β (TGF-β) is known to be a potent fibrogenic factor, which enhances the formation of glial scar after neuron inflammation, which impedes neural regeneration [13, 14]. TGF-β also increases microglia and astrocyte activation, fibronectin, and laminin deposition [15].

IL-20, a member of IL-10 family, is a pleiotropic inflammatory cytokine [16, 17] and affects multiple cell types by activating a heterodimer receptor complex: IL-20R1/IL-20R2 or IL-22R1/IL-20R2 [18]. Previous studies [19–23] have reported that IL-20 and its receptors are expressed on osteoclasts, osteoblasts, hepatocytes, rheumatoid synovial fibroblasts, proximal tubular epithelial cells, and breast cancer cells. IL-20 is involved in multiple inflammatory diseases [24] including psoriasis [16, 25, 26], atherosclerosis [24, 27], rheumatoid arthritis [20], ischemic stroke [28], acute renal failure, and chronic kidney disease [21, 29].

Although the central nervous system has been regarded as an immunologically privileged area, we previously [28, 30] reported that IL-20 was expressed in astrocytes in a rat ischemic model. IL-20 acts as a pivotal factor in fibrogenesis and is involved in renal fibrosis and liver cirrhosis through upregulating TGF-β production [31, 32]. However, the role of IL-20 in SCI remains unclear. Since the inflammatory reaction and the subsequent glial scar formation are the most detrimental pathogenesis in SCI, we hypothesized that IL-20 might be upregulated after SCI and involved in regulating astrocyte reactivation and axonal regeneration and was superimposed upon the secondary injury. Therefore, we were aimed to investigate the molecular mechanism that IL-20 was involved in the pathogenesis of SCI and explore the efficacy of IL-20 blockade in the rat model with SCI.

## Methods

### Cell culture

Rat adrenal pheochromocytoma cell line PC-12 was kindly provided by Professor Ming-Shaung Ju (National Cheng Kung University, Taiwan). PC-12 cells were maintained in RPMI-1640 medium (Hyclone, Logan, UT) supplemented with 10% (v/v) FBS (Hyclone), 100 U/ml penicillin, and 100 mg/ml streptomycin (Hyclone) and kept at 37 °C in a 5% CO_2_/95% air atmosphere. Fifty nanograms per milliliter nerve growth factor (NGF) (R&D systems, USA) was added to differentiate PC-12 cells. The human astrocyte cell line HA was purchased from ScienCell Research Laboratories (Carlsbad, CA) and maintained in Astrocyte Medium (ScienCell Research Laboratories).

### Antibody preparation

Anti-IL-20 monoclonal antibody (7E) was prepared as previously described [24]. 7E was generated with standard protocols. 7E specific inhibited and neutralized IL-20’s biological function in vitro and in vivo [22, 28, 32, 33]. The molecular weight of IL-20 and anti-IL-20 mAb 7E is 15 kDa and 150 kDa, respectively. Therefore, the monoclonal antibody 7E used to neutralize IL-20 at a 10:1 concentration (7E: IL-20) was performed to demonstrate the specific activity of IL-20 in vitro.

### Immunocytochemical staining

Cells were fixed in 3.7% paraformaldehyde and then permeabilized using phosphate-buffered saline (PBS) with 0.1% Triton X-100. The cells were blocked by immersing them in antibody diluent with background reducing components (Dakocytomation, Carpinteria, CA) and then incubated with primary antibodies: anti-IL-20 (7E), anti-IL-20R1, anti-IL-20R2, and anti-IL-22R1 (R&D systems) in blocking reagent. Immunocytochemical analysis was done using a chromogen kit (Romulin AEC Chromogen Kit; Biocare Medical, Walnut Creek, CA) and counterstained with Mayer’s hematoxylin (J. T. Baker, Phillipsburg, NJ). Images were taken using a scanning confocal laser microscopy (Olympus FV1000).

### Quantitative real-time polymerase chain reaction

Total RNA was isolated. Reverse transcription was performed with reverse transcriptase (Thermo Scientific, Rockford, IL). Complementary DNA (cDNA) from expressed mRNA was then amplified on a thermocycler (LightCycler 480; Roche Diagnostics, Indianapolis, IN), with SYBR Green I (Roche Diagnostics) as the interaction agent. The quantitative analysis of mRNA was normalized with GAPDH as the housekeeping gene. Relative multiples of change in mRNA expression were determined by calculating 2^−ΔΔCt^.

### Wound-healing assay

HA cells were seeded in a 24-well plate for 24 h and then scratched a wound through the entire center of the well using a sterilized disposable 200-μl pipette tip. Cells were washed 3 times with PBS, then serum-free medium was added. Cells were incubated with IL-20 (200 ng/ml), 7E (2 μg/ml), or IL-20 (200 ng/ml) plus 7E (2 μg/ml) for 24 h. Wound photographs were taken 24 h after treatment. Wound closure was quantified by measuring the area remaining to be immigrated using the ImageJ software (NIH Image software).

### Cell viability assay

NGF-differentiated PC-12 cells were incubated with IL-20, 7E, or IL-20 plus 7E for 72 h. The cells were then incubated with 0.5 mg/ml of methylthiazol tetrazolium (MTT) (Sigma-Aldrich, St. Louis, MO) for 3 h. The supernatant was aspirated, and the MTT-formazan crystals were dissolved in dimethylsulfoxide (DMSO) (Sigma-Aldrich). Absorbance was determined at 550 nm.

### Neurite outgrowth assay

PC-12 cells were seeded on poly-l-lysine-coated plates with maintained medium. After 24 h, cells were changed to differentiation medium containing NGF (50 ng/ml) and treated with or without IL-20 (200 ng/ml) for 72 h. Cells were then fixed and stained with anti-β-tubulin antibody. The length of the longest neurite of β-tubulin-positive cells was measured by using the ImageJ 1.42 software (NIH Image software).

### Animals

Adult female Sprague Dawley rats (220–250 g) were purchased from BioLASCO Taiwan Co. (Taipei, Taiwan) and kept on a 12 h light-dark cycle at 22 ± 2 °C. All surgical intervention, preoperative care, and treatment of all animals were in strict accordance with Institutional Animal Care and approved by the Animal Ethics Committee of National Cheng Kung University in Tainan, Taiwan (IACUC approval no. 105043). All methods were carried out in accordance with the approved guidelines. Every effort was made to minimize animal suffering and to reduce the number of animals used.

### Moderate-contusive SCI rat model

The surgical procedures for rats subjected to SCI were well described in our previous study [34]. In brief, moderate-contusive SCI was conducted by dropping a 10-g rod from 25 mm onto the exposed dura of the T9 vertebrae at the spinal cord level. After injury, rats were placed on a heating pad to maintain body temperature at 37 °C until recovery from anesthesia. Each rat was housed individually with freely available water and rodent chow. The urinary bladder of each rat was emptied 3 times per day until the bladder reflex was reestablished. To determine the levels of IL-20 and TGF-β1 after SCI, rats with/without SCI were analyzed at 1 h, 6 h, 12 h, 1 day, 3 days, 5 days, and 7 days post-SCI using immunohistochemical (IHC) staining, immunofluorescence (IF) staining, and western blotting. In a separate experiment, SCI rats were treated with/without anti-IL-20 mAb 7E (5–10 mg/kg) to evaluate the optimal dosage of anti-IL-20 mAb 7E in the SCI rat model. The spinal cord tissues were collected and analyzed at 3 days post-SCI. To evaluate the therapeutic potential of 7E in the SCI rat model, SCI rats treated with/without anti-IL-20 mAb were tested for functional outcome through sensory and motor functions at 28 days post-SCI. The spinal cold tissues were collected for histological examination. All of the animal studies were conducted in a blinded manner. All the surgical procedures were performed by the neurosurgeon, Dr. Jung-Shun Lee (first author). Afterwards, the rats were delivered to a research associate who was not involved in the surgery, but responsible for the postoperative care until rats were recovered from the anesthesia, and the anti-IL-20 mAb 7E/placebo were delivered into the rats.

### Western blotting

The spinal cords were freshly collected and homogenized in tissue lysis buffer. The lysates were centrifuged at 12,000 rpm for 15 min at 4 °C, and the supernatant was collected and stored at − 80 °C. The Bradford method (Bradford, 1976) was performed to quantitate protein concentration. SDS-PAGE electrophoresis was performed by using a 12% polyacrylamide gel and 30 μg/well protein, and separated protein was then transferred for 45 min onto a nitrocellulose membrane. The membrane was blocked with 5% non-fat milk in TBST (20 mM Tris base, 130 mM NaCl, 0.1% Tween-20) and then incubated overnight at 4 °C with primary antibodies: anti-IL-20, anti-TGF-β1 (Cell Signaling), and anti-β-actin (Proteintech®). After primary antibody incubation, the membrane was washed with TBST. Following the wash steps, the membrane was incubated with species-specific horseradish peroxidase-labeled (HRP) secondary antibody for 1 h at room temperature. Following the final wash steps, the membrane was developed with enhanced chemiluminescent substrate and visualized on film (FUJI MEDICAL X-RAY FILM, FUJIFILM, Japan). The protein signals were digitalized by a scanner and analyzed by the Image-pro plus 4.5.1 software.

### Preparing for histological examination

Ice-cold normal saline followed by 4% paraformaldehyde in 0.1 M phosphate buffer at pH 7.4 was used for transcardially perfusion. The spinal cord tissues were collected and postfixed in 4% PFA at 4 °C for 4 h. The spinal cord was first dissected into a 10-mm segment, centered over the lesion epicenter, was further dissected, immersed in PBS with 30% sucrose for cryoprotection at room temperature for 48 h, and embedded in optimal cutting temperature compound in liquid nitrogen. The cryosections of the spinal cord were cut transversely at 20 μm in a cryostat (LEICA CM1950) at − 20 °C. Ten sections at intervals of 480 μm were sampled in a rostral-to-caudal order from each animal, mounted onto frosted slides (Matsunami, Japan), and then stored at − 20 °C for further analysis. On each slide, we selected rostrally the first sample section that manifested the least injury as the prelesion section, whereas the one with the most severe tissue damage was defined as the section of the lesion epicenter.

### Immunohistochemical and immunofluorescence staining

Cryosections of the spinal cord tissue were rinsed with 0.01 M PBS, and then blocked with 5% normal goat serum, 0.1% bovine serum albumin, and 0.2% Triton-X 100 in 0.01 M PBS before applying primary antibody. The following primary antibodies were used: mouse anti-IL-20 (1:800), mouse anti-Neuronal Nuclei (1:1200, Millipore, MAB377), rabbit anti-Oligo2 (1:4000, Chemicon, AB9610), rabbit anti-GFAP (1:1000, Dako, Z0334), rabbit anti-Iba1 (1:500, Wako, 019-19741), and mouse anti-CSPG (1:200, Sigma, 115-065-075). Target signals were visualized by using mouse/rabbit polydetector HRP with DAB kit (Bio SB, BSB0205) or fluorescence-conjugated secondary antibodies.

### Luxol fast blue staining

Spinal cord myelination was evaluated via luxol fast blue (LFB) staining at day 28 post-SCI. Serial spinal cord transverse cryosections (20-μm thick, 400-μm intervals) were rinsed with 100% and 95% ethanol. Following the wash steps, tissues were stained with LFB staining solution (Sigma, 0.1% LFB in 95% ethanol with 0.5% acetic acid) at 60 °C. Cryosections were rinsed in 95% ethanol and differentiated in 0.05% lithium chloride solution. The LFB-positive areas were calculated as LFB staining area/total section area × 100% by the ImageJ software.

### Behavioral examination

Functional outcome assessments were divided into locomotor function and sensory conductive function. Locomotor functions were recorded weekly for 4 weeks, using the Basso, Beattie, and Bresnahan (BBB) scale by two blinded observers. The procedures for assessing locomotor function were well described in our previous study [34]. Sensory function was evaluated by cortical somatosensory evoked potentials (CSEPs) at day 28 post-SCI. The cortical recordings used a cathode, which was a 0.6-mm threaded screw placed 3.0-mm lateral and 2.0-mm posterior to the bregma, and an anode, which was another screw inserted 2.0-mm posterior to the lambda. The peripheral needle electrodes were inserted subcutaneously at the medial ankle to the tibial nerve, just medial to both ankles for hind leg CSEP stimulation and at the forepaw for foreleg CSEP stimulation.

### Experimental design and statistical analysis

The statistical method used in each dataset is indicated in the figure legends. Data from two groups were compared using Student’s *t* test. Data from three or more groups were compared using one-way ANOVA followed by Bonferroni’s post hoc test. The continuous variables were expressed as mean ± standard deviation. A *p* value < 0.05 was considered statistically significant. All statistical analyses were carried out using Prism 8th edition.

## Results

### Upregulation of IL-20 after spinal cord injury

**Fig. 1:**
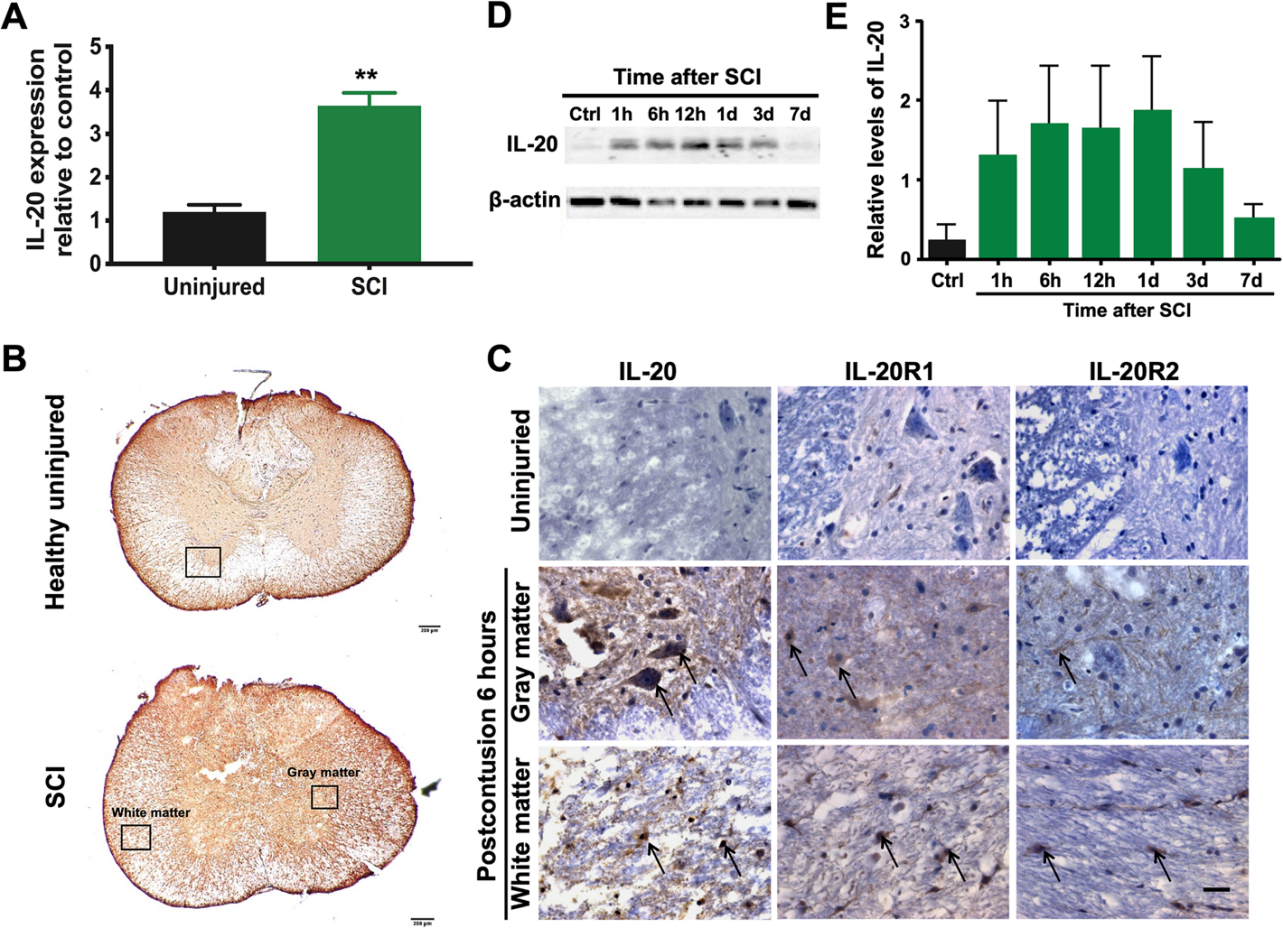
Upregulation of IL-20 after spinal cord injury (SCI). **a** Spinal cord tissues from healthy rats (uninjured; *N* = 4) and SCI rats (*N* = 6) were collected at 3 days post-SCI. Total RNA was isolated and the transcripts of IL-20 were measured using RT-qPCR with specific primers. GAPDH was an internal control. **P < 0.01 compared with the healthy uninjured controls. Data are expressed as mean ± SD. **b** Spinal cord sections obtained from healthy uninjured rats (*N* = 5) and SCI rats (*N* = 5) at 6 h after the initial injury. Scale bars = 200 μm. **c** Spinal cord tissue samples were stained with anti-IL-20 mAb using immunohistochemical staining. Staining for IL-20 was positive in the injured spinal cord, not only in the gray matter, but also in the white matter. Scale bars = 500 μm. **d** Spinal cord tissue from healthy control rats (*N* = 5) and SCI rats (*N* = 5; each time point) were collected at the indicated time points post-SCI. Tissue lysates were analyzed through immunoblotting with specific antibodies against IL-20. β-actin was an internal control. **e** Relative levels of IL-20 quantified by densitometric analysis using ImageJ software. Data are expressed as mean ± SD and are representative of three independent experiments

To examine the involvement of IL-20 in the pathogenesis of SCI, we analyzed the expression of IL-20 in SCI rats and compared it to that of healthy, uninjured control rats. RT-qPCR showed that IL-20 was upregulated in the spinal cord of SCI rats compared to healthy control rats (Fig. 1a). Immunohistochemical (IHC) staining showed that IL-20 and its receptors (IL-20R1 and IL-20R2) were remarkably stained in the injured spinal cord, not only in the gray matter but also in the white matter at 6 h post-SCI (Fig. 1b, c). We performed western blotting to clarify the expression trend of IL-20 after SCI. The temporal expression of IL-20 protein elevated quickly, with obvious upregulation at 1 h after injury, and the expression was still detectable at 7 days post-SCI (Fig. 1d, e).

**Fig. 2:**
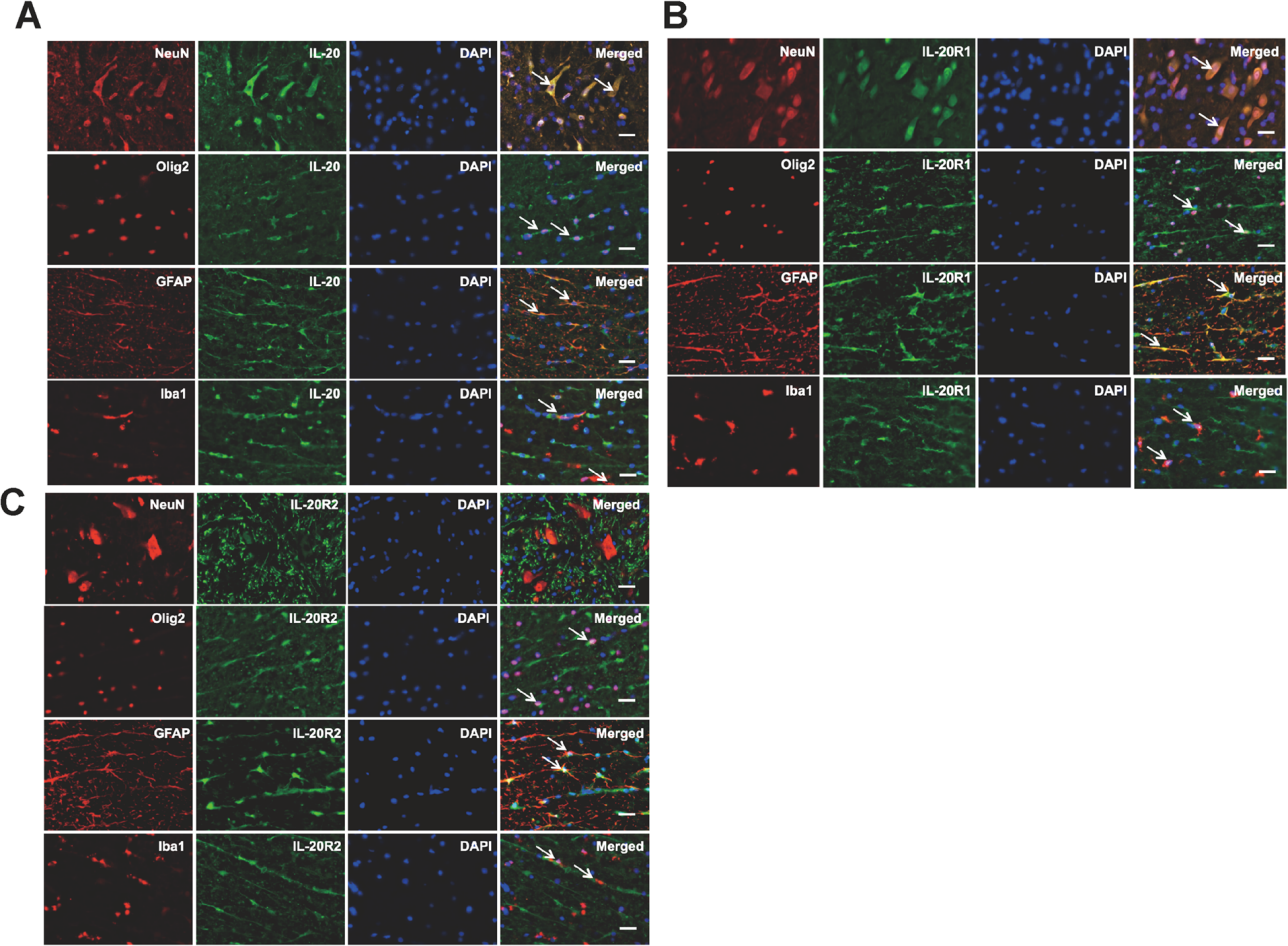
Expression of IL-20, IL-20R1, and IL-20R2 in spinal cord tissues after SCI. The transverse sections around the interface between gray and white matters of the spinal cord obtained from SCI rats (*N* = 5). **a** Double immunofluorescence staining of IL-20 (green) with markers for specific neural cell types (red) including NeuN (neurons), Olig2 (oligodendrocytes), GFAP (astrocytes), and Iba1 (microglia). Nuclei were counterstained with DAPI (blue). Co-localization of IL-20 with each cellular marker appears yellow in the merged image. Scale bars = 500 μm. **b**, **c** Double immunofluorescence staining of IL-20R1 (green) or IL-20R2 (green) with markers for specific neural cell types (red) as described above. Co-localization of IL-20R1 or IL-20R2 with each cellular marker appears yellow in the merged image. Scale bars = 500 μm. NeuN, neuronal nuclei; Olig2, oligodendrocyte transcription factor 2; GFAP, glial fibrillary acidic protein; Iba1, ionized calcium-binding adapter molecule 1. Data from one representative experiment of three independent experiments is shown

To further determine the possible cellular sources and the target cells of IL-20 in the spinal cord, the transverse sections around the interface between gray and white matters of the anterior column and anterior horn were labeled with antibodies specific to IL-20, IL-20R1, IL-20R2, glial fibrillary acidic protein (GFAP; astrocyte maker), neuronal nuclei (NeuN: neuron marker), oligodendrocyte transcription factor 2 (Olig2; oligodendrocyte marker), and ionized calcium-binding adapter molecule 1 (Iba1; microglia marker). Double immunofluorescence staining revealed that IL-20 was expressed in neurons, astrocytes, oligodendrocytes, and microglia (Fig. 2a). In addition, these cells expressed both IL-20R1 and IL-20R2, with the exception of neurons, which only expressed IL-20R1 (Fig. 2b, c). These results indicate that the IL-20 is involved in the pathogenesis of traumatic SCI.

### IL-20 enhanced astrocyte reactivation and migration of HA cells

**Fig. 3:**
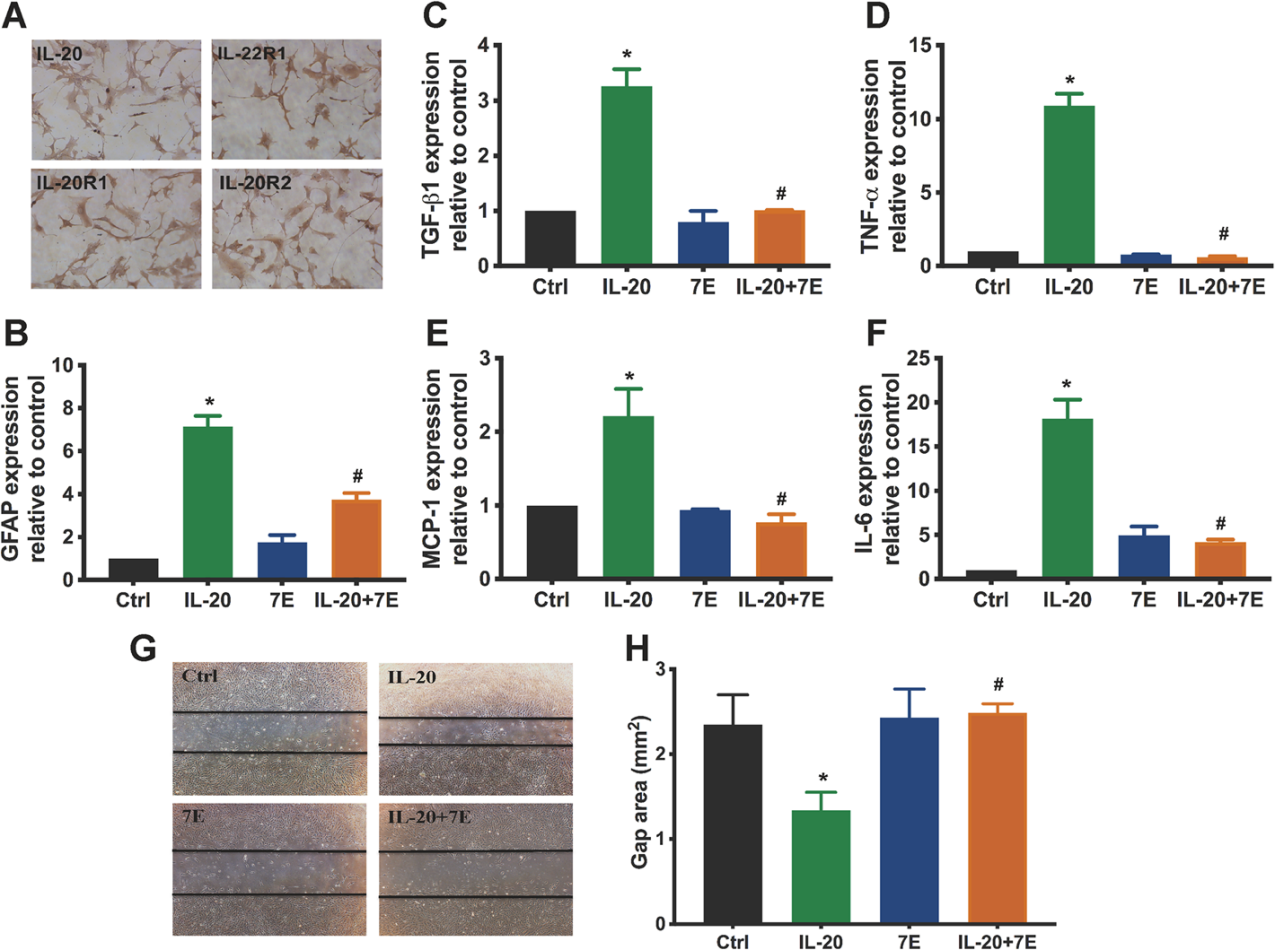
IL-20 promoted astrocyte activation in HA cell line. **a** Expression of IL-20 and its receptors (IL-20R1, IL-20R2, and IL-22R1) in HA cells was analyzed using immunocytochemistry staining. Data from one representative experiment of three independent experiments is shown. **b**–**f** HA cells were treated with IL-20 (200 ng/ml), 7E (2 μg/ml), or IL-20 (200 ng/ml) plus 7E (2 μg/ml) for 4 h. Total RNA was isolated and the transcripts of GFAP, TGF-β1, TNF-α, MCP-1, and IL-6 were analyzed using RT-qPCR with specific primers. GAPDH was an internal control. *P < 0.05 compared with the untreated controls (Ctrl). ^#^*P* < 0.05 compared with the IL-20-treated group. Data are expressed as mean ± SD and are representative of three independent experiments performed in triplicate. **g**, **h** Cell migration was evaluated using a wound-healing assay for 24 h. Wound closure was quantified by measuring the area remaining to be immigrated using ImageJ software. *P < 0.05 compared with the untreated controls (Ctrl). ^#^*P* < 0.05 compared with the IL-20-treated group. Data are expressed as mean ± SD and are representative of three independent experiments performed in triplicate

Based on our data from SCI rats, astrocytes are target cells for IL-20. Therefore, we used a human astrocyte HA cell line to explore the possible effects of IL-20. Immunocytochemical staining showed that IL-20 and its three receptors (IL-20R1, IL-20R2, and IL-22R1) were all expressed in HA cells (Fig 3a). Upregulation of GFAP expression is one of the main characteristics of the astrocytic reaction [35]. To identify a possible role for IL-20 in astrocyte activation, GFAP mRNA expression was analyzed by RT-qPCR. HA cells treated with IL-20 showed induction of GFAP at levels greater than 7-fold (Fig. 3b). We further clarified whether IL-20 directly affects other cytokine regulation in HA cells. RT-qPCR showed that the expression of TGF-β1, TNF-α, MCP-1, and IL-6 transcripts was significantly upregulated by IL-20, which was inhibited by anti-IL-20 mAb 7E (Fig. 3c–f). To characterize astrocyte activity in response to IL-20, we performed a MTT assay and found that IL-20 did not affect cell proliferation in HA cells (data not shown). However, a wound healing assay showed that cell migration ability was significantly increased in IL-20-treated HA cells compared with untreated controls (Fig. 3g–h).

### IL-20 affected cell viability and inhibited neurite outgrowth in PC-12 cells

**Fig. 4:**
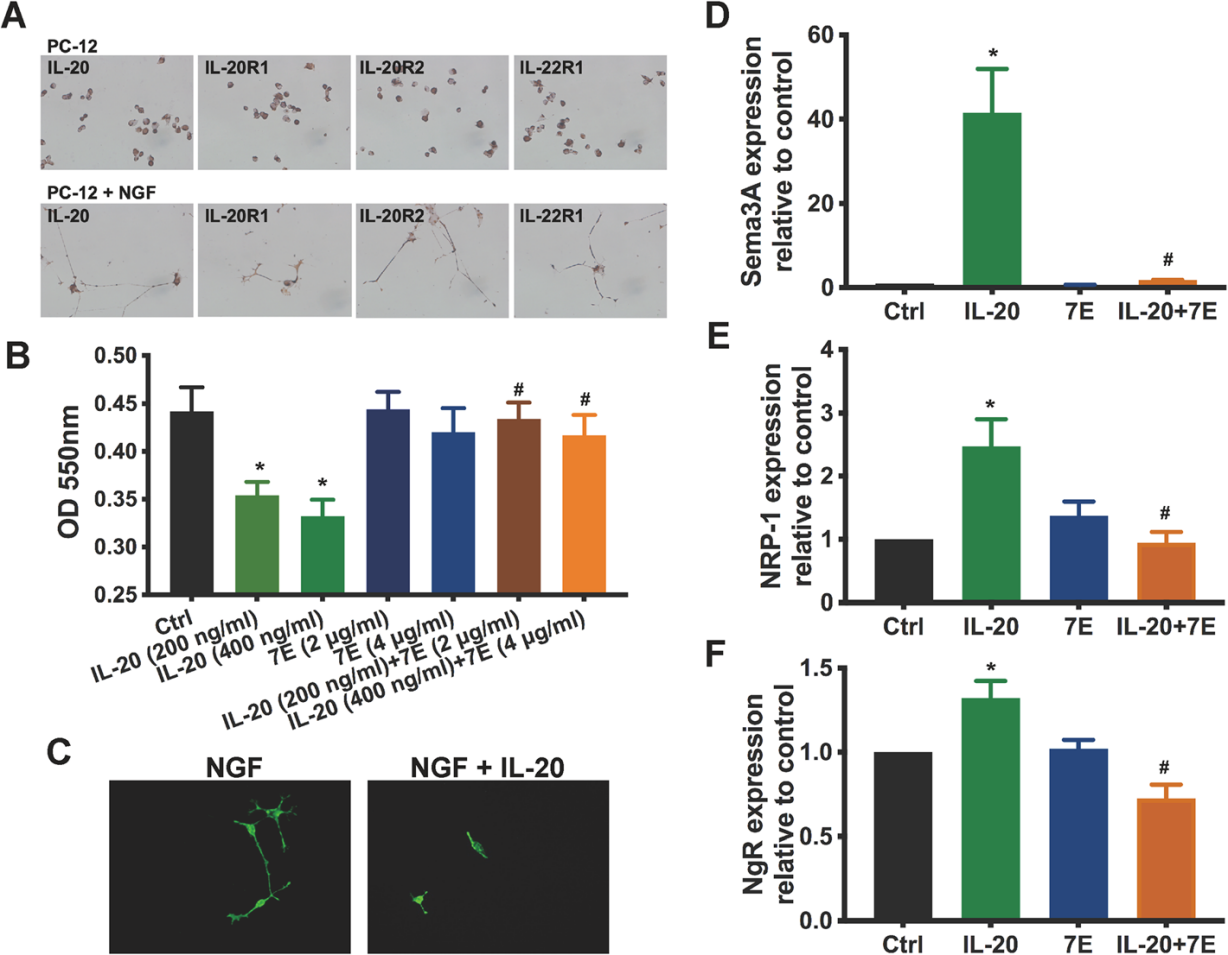
IL-20 inhibited axonal regeneration. **a** Expression of IL-20 and its receptors (IL-20R1, IL-20R2, and IL-22R1) in PC-12 cells and nerve growth factor (NGF)-differentiated PC-12 cells were analyzed using immunocytochemistry staining. Data from one representative experiment of three independent experiments is shown. **b** NGF-differentiated PC-12 cells were incubated with IL-20, 7E, or IL-20 plus 7E for 72 h. Cell viability was determined using the MTT assay. *P < 0.05 compared with the untreated controls (Ctrl). ^#^*P* < 0.05 compared with the corresponding controls. Data are expressed as mean ± SD and are representative of three independent experiments performed in quadruplicate. **c** PC-12 cells were treated with NGF or with NGF plus IL-20 (200 ng/ml). Immunofluorescence staining was performed using anti-β-tubulin mAb (green) to mark neurite outgrowth. Data from one representative experiment of three independent experiments is shown. **d** NGF-differentiated PC-12 cells were treated with IL-20 (200 ng/ml), 7E (2 μg/ml), or IL-20 (200 ng/ml) plus 7E (2 μg/ml) for 6 h. Total RNA was isolated, and the transcripts of Sema3A, NRP-1, and NgR were analyzed using RT-qPCR with specific primers. GAPDH was an internal control. *P < 0.05 compared with the untreated controls (Ctrl). ^#^*P* < 0.05 compared with IL-20-treated group. Data are expressed as mean ± SD and are representative of three independent experiments performed in quadruplicate

To explore the role of IL-20 in axonal regeneration, we analyzed the expression of IL-20 and its receptors in PC-12 cells, a rat adrenal pheochromocytoma cell line that responds reversibly to nerve growth factor (NGF) by induction of the neuronal phenotype. Immunocytochemical staining showed that IL-20 and its receptors were expressed in PC-12 cells and NGF-derived differentiated neuron-like PC-12 cells (Fig. 4a). We performed a MTT assay to examine the effects of IL-20 on cell death and inhibition of proliferation in NGF-derived differentiated PC-12 cells. IL-20 affected cell viability in NGF-derived differentiated PC-12 cells, which was inhibited by anti-IL-20 mAb 7E (Fig. 4b). These data raise the possibility that IL-20 may directly affect neurite outgrowth. To test this hypothesis, PC-12 cells were incubated with or without IL-20 and cultured in differentiation medium containing NGF to evaluate the effect of IL-20 on neurite outgrowth in vitro. Immunofluorescence staining of anti-β-tubulin was performed to visualize neurite outgrowth. IL-20 significantly inhibited NGF-derived neurite outgrowth (Fig. 4c). In addition, RT-qPCR showed that IL-20 upregulated the expression of Sema3A and NRP-1 in NGF-derived differentiated PC-12 cells (Fig. 4d, e). IL-20 also induced the expression of Nogo receptor (NgR), which acts as an axon outgrowth inhibition receptor (Fig. 4f). These results suggest that IL-20 is an important regulator for axonal regeneration.

### Anti-IL-20 mAb 7E reduced the inflammatory response in SCI rats

**Fig. 5.**
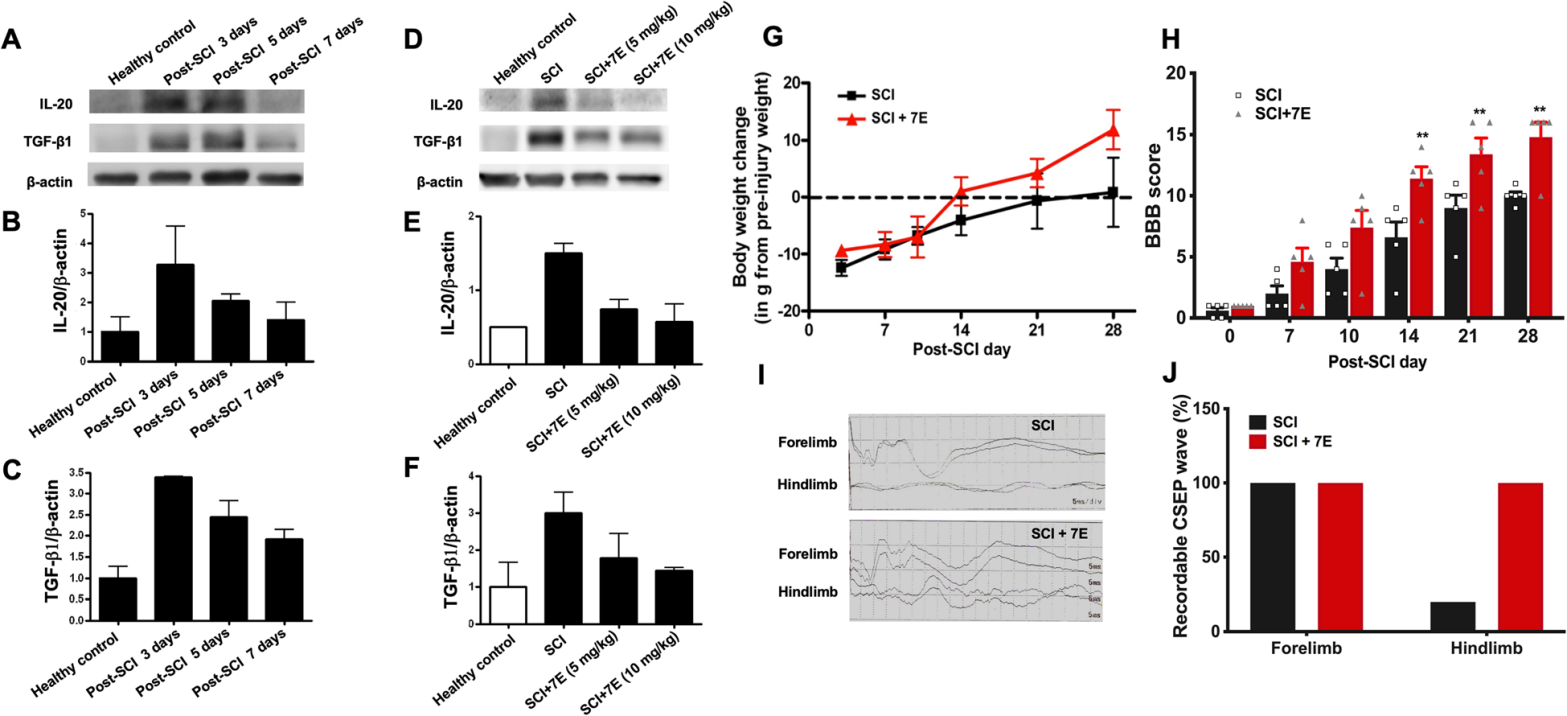
Anti-IL-20 mAb 7E improved motor function and sensory function in SCI rats. **a** Spinal cord tissues from healthy control rats (*N* = 4) and SCI rats were collected at 3 days, 5 days, and 7 days post-SCI (*N* = 4; each time point). Tissue lysates were analyzed through immunoblotting with specific antibodies against IL-20 and TGF-β1. β-actin was an internal control. **b**, **c** Relative levels of IL-20 and TGF-β1 quantified by densitometric analysis using ImageJ software. Data are expressed as mean ± SD and are representative of three independent experiments. **d** Spinal cord tissues from healthy control and SCI rats treated with anti-IL-20 mAb 7E (5–10 mg/kg) were analyzed at 3 days post-SCI through immunoblotting with specific antibodies against IL-20 and TGF-β1 (*N* = 4/group). β-actin was an internal control. **e**, **f** Relative levels of IL-20 and TGF-β1 quantified by densitometric analysis using ImageJ software. Data are expressed as mean ± SD and are representative of three independent experiments. **g** SCI rats were treated with anti-IL-20 mAb 7E (10 mg/kg), and body weight was measured weekly (*N* = 5/group). **h** The Basso, Bresnahan, and Beattie (BBB) scoring method was applied to evaluate motor function in SCI control rats (*N* = 5/group) and SCI rats (*N* = 5/group) treated with anti-IL-20 mAb 7E (10 mg/kg) weekly post-SCI. **P < 0.01 compared with the SCI rats. Data are expressed as mean ± SD. Data from one representative experiment of three independent experiments is shown. **i**, **j** The cortical somatosensory-evoked potential (CSEP) waves were recorded to represent sensory function in SCI control rats and SCI rats treated with anti-IL-20 mAb 7E (10 mg/kg) at 28 days post-SCI (*N* = 5/group). Data from one representative experiment of three independent experiments is shown

We further assessed the efficacy of IL-20 blockade in rats after SCI. TGF-β is involved in nerve injury-induced inflammation and tissue repair and worsens the outcome in SCI. We generated a mouse anti-human IL-20 monoclonal antibody 7E and demonstrated its therapeutic potential because it could inhibit IL-20 in vitro and in vivo [22, 28, 32, 33]. We first attempted to determine the optimal therapeutic dosage of anti-IL-20 mAb; the temporal expression of IL-20 and TGF-β1 were measured at 3, 5, and 7 days post-SCI. Western blotting showed that the expression of both IL-20 and TGF-β1 were significantly elevated at 3 to 5 days post-SCI (Fig. 5a–c). Therefore, 3 days post-SCI was set as the checkpoint to analyze the optimal dosage of anti-IL-20 mAb. Western blotting showed that anti-IL-20 mAb treatment (5 mg/kg or 10 mg/kg) reduced both IL-20 and TGF-β1 expression after SCI (Fig. 5d–f). We also observed no unexpected mortalities or morbidities related to antibody treatment (data not shown). A higher dosage of anti-IL-20 mAb (10 mg/kg) resulted in better inhibition of inflammatory protein expression; therefore, the optimal therapeutic dosage was set at 10 mg/kg in the following in vivo studies.

### Anti-IL-20 mAb 7E treatment improved both motor and sensory functions

In a SCI rat model, anti-IL-20 mAb treatment resulted in a return to pre-injury body weight at 14 days post-SCI, followed by additional body weight gain, while the control group did not return to the baseline body weight until 28 days post-SCI (Fig. 5g). To evaluate the beneficial effects of anti-IL-20 mAb for motor function improvement in SCI rats, the Basso, Bresnahan, and Beattie (BBB) scoring method was applied to evaluate motor function weekly in control rats and rats treated with anti-IL-20 mAb 7E (10 mg/kg) after SCI. The mean BBB scores in the IL-20 mAb-treated group were higher than that in the control group at 7 days post-SCI (Fig. 5h), which indicated that IL-20 mAb treatment slightly improved motor function. Moreover, the IL-20 mAb-treated group exhibited significantly better motor function than the control group from 14 days post-SCI until the end of the study (28 days post-SCI) (Fig. 5h).

To examine sensory function improvement, cortical somatosensory evoked potential (CSEP) waves were recorded to represent sensory function in SCI control rats and in SCI rats treated with IL-20 mAb 7E (10 mg/kg). For sensory function, the forelimb CSEP represented the uninjured spinal cord and was recorded as an internal control. The forelimb CSEP waves in both groups were recordable (Fig. 5i). By contrast, most of the hindlimb CSEP waves in the control group were diminished (4/5; 20% detectable), while all rats in IL-20 mAb-treated group showed recordable waveform (5/5; 100% detectable) (Fig. 5i, j).

### Anti-IL-20 mAb 7E treatment improved spinal cord tissue preservation and reduced glial scar formation

**Fig. 6:**
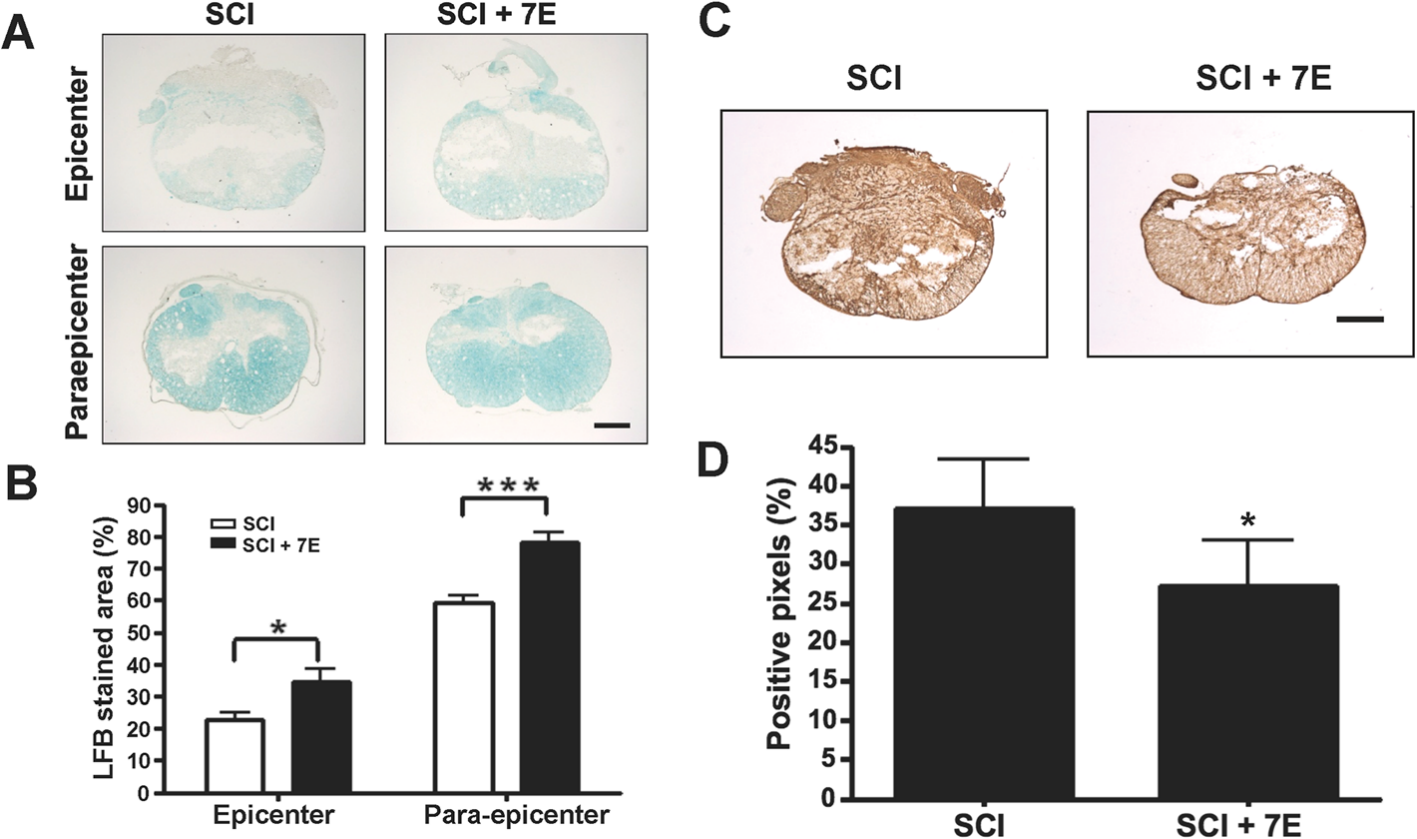
Anti-IL-20 mAb 7E improved spinal cord tissue preservation and reduced glial scar formation. **a** Luxol fast blue (LFB) stain was used to identify myelinated tissues in SCI control rats and in SCI rats treated with anti-IL-20 mAb 7E (10 mg/kg) at 28 days post-SCI (*N* = 5/group). Scale bars = 500 μm. **b** Quantification of LFB stained areas. *P < 0.05, ***P < 0.001 compared with the SCI rats. Data are expressed as mean ± SD. Data from one representative experiment of three independent experiments is shown. **c** At 28 days post-SCI, spinal cord tissue samples from SCI control rats and from SCI rats treated with anti-IL-20 mAb 7E (10 mg/kg) were stained with anti-chondroitin sulfate proteoglycan **(**CSPG) mAb to evaluate glial scar formation (*N* = 5/group). Scale bars = 500 μm. **d** Quantification of CSPG-positive stained areas. *P < 0.05 compared with the SCI rats. Data are expressed as mean ± SD. Data from one representative experiment of three independent experiments is shown

Many axons are covered by sheaths of an insulating substance called myelin. Loss of myelin can occur with SCI and prevent effective transmission of nerve signals. Luxol fast blue (LFB) staining was performed to identify myelinated tissues. More myelinated tissues were observed in the IL-20 mAb-treated SCI rats than in the SCI control rats, especially in the para-epicenter (Fig. 6a, b). This result indicates that IL-20 mAb treatment could prevent tissue damage after SCI.

Chondroitin sulfate proteoglycans (CSPGs), the major component of glial scars, are known to impede neural sprouting after SCI. The intensity of CSPG staining was significantly decreased in the IL-20 mAb-treated SCI rats, which indicates that IL-20 mAb treatment could reduce glial scar formation (Fig. 6c, d). These results are concordant with the findings of functional improvement after IL-20 mAb treatment in SCI rats.

## Discussion

In this study, we found that IL-20 was upregulated in the spinal cord after SCI. IL-20 and its receptors were expressed in several key cell types in the spinal cord of SCI rats, including astrocytes, microglia, oligodendrocytes, and neutrons. In vitro, IL-20 enhanced astrocyte reactivation, cell migration, and cytokine regulation in HA cells. IL-20 affected cell viability and inhibited neurite outgrowth through Sema3A-/NRP-1 upregulation in PC-12 cells. In vivo, anti-IL-20 mAb reduced the IL-20-mediated inflammatory response, improved both motor and sensory functions, and also improved spinal cord tissue preservation and reduced glial scar formation. Collectively, these findings suggest that IL-20 is critical for the neuroinflammatory response and is involved in astrocyte reactivation and axonal regeneration. This profound inflammation results in severe gliosis, which inhibits functional improvements during the chronic stage of SCI.

A secondary cascade of vascular, molecular, inflammatory, and biochemical events is triggered after the initial SCI that further disrupts the functions of neural networks. These primary and secondary injury events activate astrocytes and microglia [1, 36]. IL-20 and its receptors are expressed in astrocytes, oligodendrocytes, microglia, and neurons, which indicate that IL-20 could target these cells to regulate cellular communication and inflammatory response through a paracrine or autocrine manner.

Astrocytes act as a pivotal regulator of CNS inflammatory responses, which form borders (glia limitans) that separate neural from non-neural tissue along perivascular spaces, meninges, and tissue lesions in the CNS [1, 3, 37]. IL-20 induced GFAP expression, providing evidence that IL-20 is involved in astrocyte reactivation. In addition, we also found that IL-20 promoted TGF-β1, TNF-α, MCP-1, and IL-6 expression in HA cells, which raises the possibility that IL-20 might stimulate astrocytes to produce these cytokines and disrupt homeostasis of the microenvironment in response to a vast array of CNS insults. A recent study [38] indicated that primary astrocytes isolated from murine and human are not an appreciable source of IL-20; however, IL-20 can upregulate TLR4 and IL-1β expression and induce IL-6 production to augment the inflammatory responses of astrocytes. This discrepancy in the cellular source of IL-20 might be due to the different animal models used and requires additional investigation.

Glial scar formation is a critical event in the repair response after CNS injury. Resident microglia and astrocytes are activated following injury and form a glial scar surrounding and sequestering the damaged tissue [8, 39]. Astrocyte migration is a key step in glial scar formation and is regulated by TGF-β1 [40, 41]. Our data show that IL-20 promotes astrocyte migration; meanwhile, IL-20 also stimulates TGF-β1 expression. Therefore, IL-20 might directly affect local inflammation and astrocyte migration, and indirectly affect these activities by inducing other mediators such as TGF-β1.

IL-20 signaling is activated through the signal transducer and activator of transcription 3 (STAT3) pathway [16]. Previous studies [42, 43] indicate that STAT3 is also critical for establishing the glial scar border that secludes infiltrating cells to the lesion epicenter. Activation of the TGF-β1 pathway by IL-6/STAT3 leads to aggravating fibrosis [44]. We previously [32] reported that IL-20 promotes fibrosis and extracellular matrix (ECM) deposition by upregulating TGF-β1. Thus, we speculate that IL-20 and TGF-β1 form an auto-amplification loop upon astrocyte activation, and both together are involved in depositing ECM proteins which form the fibrous scar that exacerbates tissue damage after SCI.

The glial scar at the lesion site is not only a physical barrier to axonal growth, but the reactive astrocytes show an increased inflammatory phenotype and may contribute to the failure of neuronal regeneration [45]. Our data show that IL-20 affects cell viability and inhibits neurite outgrowth in NGF-derived differentiated neuron-like PC-12 cells. Semaphorin3A (Sema3A) signaling via its receptor neuropilin-1 (NRP-1) is a critical pathway for inhibiting neuron growth [11]. We found that IL-20 upregulated Sema3A and NRP-1 expression. These results suggest that IL-20 suppresses neurite outgrowth through Sema3A/NRP-1 upregulation. Furthermore, Nogo receptor (NgR) signaling has a central role in the inhibition of axonal regeneration [9, 10]. Blocking the Nogo-A/NgR signal on neurons enables the extension of axons over the insulted zone, which is beneficial for neuron regeneration and the functional improvement of the injured CNS [46]. IL-20 regulates NgR expression, which raises another possible mechanism for IL-20 in neurite outgrowth. Whether IL-20 directly regulates Nogo-66 and myelin-associated glycoprotein (MAG), the ligand of NgR, requires additional investigation.

As neuroinflammatory responses play a pivotal role in pathogenesis and determine the outcome of traumatic SCI, numerous trials to alleviate neuroinflammation have been carried out [47–49]. Inflammation is regulated by proinflammatory cytokines including TNFα, IL-1β, and IL-6; therefore, these cytokines have been the targets for potential pharmaceutical interventions for SCI [50–52]. However, there are not yet any effective clinical therapies. Therefore, efforts to find the key inflammatory regulators or the most upstream proinflammatory cytokine are emerging, which aim to target the inflammatory cascades. In our in vivo study, anti-IL-20 mAb treatment not only inhibited IL-20, but also significantly reduced TGF-β1 production in SCI rats, which suggests that IL-20 is the upstream regulator of TGF-β1. These data are consistent with our in vitro findings. Furthermore, anti-IL-20 mAb treatment improved motor and sensory functions, enhanced spinal cord tissue preservation, and reduced glial scar formation. Therefore, IL-20 is induced in the acute early stage of SCI, and the expression persists to the subacute phase. This relatively long duration of detectable IL-20 offers a potentially wide therapeutic window.

We hypothesize a working model for the role of IL-20 in the progression of SCI as follows. After SCI, a large amount of IL-20 is expressed in neurons, astrocytes, oligodendrocytes, and microglia. IL-20 initially stimulates astrocyte reactivation and migration by inducing the expression of TGF-β1, which in turn enhances glial scar border formation. The activated astrocytes and microglia trigger an inflammatory response by producing more cytokines and chemokines to promote fibrosis and extracellular matrix (ECM) deposition, which exacerbate tissue damage. Moreover, IL-20 reduces viability of neuronal cells and inhibits neuron outgrowth through upregulation of Sema3A/NRP-1. Eventually, all of these effects contribute to irreversible neuronal loss and glial scar formation in SCI. Anti-IL-20 mAb treatment in SCI rats potently inhibits IL-20-mediated tissue destructive effects, which supports our hypothesis.

## Conclusions

In conclusion, our findings demonstrate that IL-20 is involved in the progression of SCI. IL-20 plays a critical role in the neuroinflammatory response and is an important regulator of astrocyte reactivation and axonal regeneration. Therefore, IL-20 may be a promising target for SCI treatment.

## Data Availability

All data generated or analyzed during this study are included in this published article.

## Abbreviations

SCI: Spinal cord injury
mAb: Monoclonal antibody
GFAP: Glial fibrillary acidic protein
NGF: Nerve growth factor
MAG: Myelin-associated glycoprotein
NeuN: Neuronal nuclei
Olig2: Oligodendrocyte transcription factor 2
NgR: Nogo receptor
BBB: Basso, Bresnahan, and Beattie
CSEP: Cortical somatosensory evoked potential
LFB: Luxol fast blue
CSPGs: Chondroitin sulfate proteoglycans
ECM: Extracellular matrix
Sema3A: Semaphorin3A
NRP-1: Neuropilin-1
OMgp: Oligodendrocyte-myelin glycoprotein
TNF: Tumor necrosis factor
IL: Interleukin
IHC: Immunohistochemical
Iba1: Ionized calcium-binding adapter molecule 1
MTT: Methylthiazol tetrazolium
DMSO: Dimethylsulfoxide
IF: Immunofluorescence
HRP: Horseradish peroxidase
TGF-β: Transforming growth factor-β
PBS: Phosphate-buffered saline
